# Monocyte Subsets in Schistosomiasis Patients with Periportal Fibrosis

**DOI:** 10.1155/2014/703653

**Published:** 2014-03-13

**Authors:** Jamille Souza Fernandes, Maria Ilma Araujo, Diego Mota Lopes, Robson da Paixão de Souza, Edgar M. Carvalho, Luciana Santos Cardoso

**Affiliations:** ^1^Serviço de Imunologia, Complexo Hospitalar Universitário Professor Edgard Santos, Universidade Federal da Bahia, Rua João das Botas s/n, Canela, 40110-160 Salvador, BA, Brazil; ^2^Instituto Nacional de Ciência e Tecnologia em Doenças Tropicais (INCT-DT), CNPQ/MCT, Brazil; ^3^Escola Bahiana de Medicina e Saúde Pública, 40050-420 Salvador, BA, Brazil; ^4^Departamento de Análises Clínicas e Toxicológicas, Faculdade de Farmácia, UFBA, 40170-115 Salvador, BA, Brazil

## Abstract

A major issue with *Schistosoma mansoni* infection is the development of periportal fibrosis, which is predominantly caused by the host immune response to egg antigens. Experimental studies have pointed to the participation of monocytes in the pathogenesis of liver fibrosis. The aim of this study was to characterize the subsets of monocytes in individuals with different degrees of periportal fibrosis secondary to schistosomiasis. Monocytes were classified into classical (CD14^++^CD16^−^), intermediate (CD14^++^CD16^+^), and nonclassical (CD14^+^CD16^++^). The expressions of monocyte markers and cytokines were assessed using flow cytometry. The frequency of classical monocytes was higher than the other subsets. The expression of HLA-DR, IL-6, TNF-**α**, and TGF-**β** was higher in monocytes from individuals with moderate to severe fibrosis as compared to other groups. Although no differences were observed in receptors expression (IL-4R and IL-10R) between groups of patients, the expression of IL-12 was lower in monocytes from individuals with moderate to severe fibrosis, suggesting a protective role of this cytokine in the development of fibrosis. Our data support the hypothesis that the three different monocyte populations participate in the immunopathogenesis of periportal fibrosis, since they express high levels of proinflammatory and profibrotic cytokines and low levels of regulatory markers.

## 1. Introduction

Schistosomiasis is a chronic and debilitating disease that affects over 200 million people worldwide and it is estimated that 700 million people live in areas at risk of infection [1, 2]. It is a disease of particular socioeconomic and public health importance, since it is prevalent in tropical and subtropical areas. In Brazil, it is estimated that about 2.5 to 7 million people are infected with* S. mansoni* and 25 million live in areas at risk of infection [3, 4]. The liver pathology of* Schistosoma mansoni* infection results from the host immune response to parasite antigens from* S. mansoni* eggs that become trapped in the portal venous system [5–7]. The granulomas formed around the egg act as barriers which prevent the dispersion of* S. mansoni* egg antigens. However, with the continuous arrival of eggs, the intense inflammatory process evolves to severe fibrosis. The liver pathology leads to the interruption of normal blood flow in the venous system of the sinusoids, resulting in portal hypertension, hepatosplenomegaly, esophageal, and gastric varices, which can lead to bleeding and even death [8, 9]. This severe form of the disease occurs in about five percent of infected subjects living in endemic areas [10, 11].

Mononuclear cells are involved in the pathogenesis of chronic liver diseases, especially those associated with fibrogenesis. Monocytes, for instance, participate in the development of fibrosis through various mechanisms including secretion of cytokines and generation of products related to oxidative stress [12]. Depending on their differentiation state and local signals, monocytes and macrophages are capable of secreting a variety of growth factors and proinflammatory, profibrotic, and anti-inflammatory cytokines [13]. Recently, human monocytes were classified into three subpopulations according to the expression of CD14 and CD16: classical (CD14^++^CD16^−^), intermediate (CD14^++^CD16^+^), and nonclassical (CD14^+^CD16^++^) [14]. The study of Liaskou et al. showed evidence implicating the intermediate monocytes in the pathogenesis of liver fibrosis caused by chronic inflammatory diseases [15]. However, to our knowledge no studies have characterized these monocyte profiles in individuals with periportal fibrosis secondary to schistosomiasis. Thus, in this study we aimed to characterize monocytes subpopulations regarding their status of activation and expression of proinflammatory, antifibrotic, profibrotic, and regulatory molecules in individuals with different degrees of periportal fibrosis.

## 2. Methodology

### 2.1. Study Design and the Endemic Area

This study was carried out in an endemic area for schistosomiasis named Água Preta, in the state of Bahia, Brazil. Água Preta is located 280 km south of Salvador, the capital of the state of Bahia. It is composed of a residential area in the center of the village and some surrounding farms. Approximately 800 individuals live in the community. They live in poor sanitary conditions and agriculture is the predominant occupation. There is one river in this region that is used for bathing, washing clothes and utensils, and leisure, exposing the residents to high risk of* S. mansoni *infection [16]. Cross-sectional parasitological surveys using Kato-Katz [17] and sedimentation techniques were conducted on three different stool samples. The inclusion criteria for this study were individuals from endemic areas who have at least one positive parasitological exam for* S. mansoni*. From the 537 individuals who agreed to participate in this study 334 were infected with* S. mansoni* (62.5%). The frequency of other helminthic infections was 43.4% for* Trichuris trichiura*, 37.4% for* Ascaris lumbricoides*, 33.7% for hookworms, and 3.5% for* Strongyloides stercoralis*. From 334 individuals who were infected with* S. mansoni*, 220 agreed to perform abdominal ultrasound, in order to determine the degree of periportal fibrosis. They also agreed to donate blood for the study of the immunological response. For this particular aim, we analyzed patients of both gender, 10 to 60 years old. Seventeen patients with grade 0 (without fibrosis), fifteen patients with grade I (incipient fibrosis), and eight with fibrosis grades II and III (moderate and severe fibrosis) were included. We had difficulty in finding patients with advanced stages of periportal fibrosis in the region of the study; only eight patients with this condition met the inclusion criteria.

Individuals under ten years old were not included, due to a low probability of developing periportal fibrosis [18], and individuals older than 60 years, due to potential senescence of the immune system. We also did not include individuals with positive serology for HIV, HTLV-1, or hepatitis virus types B and C; all of which are conditions that could interfere with the immunological response.

### 2.2. Ultrasound Examination

Abdominal ultrasound (USG) was performed using the Quantum 2000 Siemens and Elegra Siemens ultrasound with a convex transductor of 3.5–5.0 Mhz. Liver span was measured in the midclavicular line. The liver was also examined for smoothness of surface, echogenicity, and posterior attenuation of the sound beam and portal vein diameter outside the liver midway between its entrance into the portal hepatic vein and its first bifurcation in the liver. Periportal fibrosis was characterized as multiple diffuse echogenic areas. Grading of periportal fibrosis was determined by the mean total thickness of four portal tracts after the first division from the right and left branches of portal vein (PT1) as follows: degree 0, mean thickness <3 mm; degree I, mean thickness 3 to 5 mm; degree II, mean thickness >5 to 7 mm; and degree III, mean thickness >7 mm [19]. We decided to use the Cairo's classification because we have performed previous studies using these parameters and because the physicians who have performed the USG in our studies are very well familiar with this classification [18, 20]. The scores of periportal fibrosis were grouped according to severity: degree 0 individuals were those without periportal fibrosis, degree I individuals those with incipient periportal fibrosis, and individuals with moderate to severe periportal fibrosis were patients with degrees II and III [21]. The analysis of immune response included 17 individuals without periportal fibrosis, 15 individuals with incipient fibrosis, and 8 individuals with moderate to severe forms of the disease, characterized by grade II (*n* = 06) and III (*n* = 02) (Table 1).

### 2.3. Features of the Studied Subject

The features of studied individuals are shown in Table 1. The mean age of patients with moderate to severe fibrosis was higher (46 ± 14 years) compared to individuals without fibrosis (30 ± 13 years; *P* < 0.05). There were no significant differences in gender distribution or in parasite burden among groups. The sizes of the liver and spleen were also evaluated in this study. There was no significant difference in liver size among groups. However, in the group of individuals with moderate to severe fibrosis, spleen size was higher as compared to the group of individuals with incipient fibrosis and without fibrosis (*P* < 0.05). Additionally, the diameter of the portal vein was higher in subjects with moderate to severe fibrosis when compared with individuals without fibrosis (Table 1).

### 2.4. Cell Culture

Peripheral blood mononuclear cells (PBMCs) were isolated using Ficoll-Hypaque gradient sedimentation and adjusted to a concentration of 3 × 10^5^/mL in RPMI 1640 medium containing 10% normal human serum (AB positive and heat inactivated), 100 U/mL of penicillin, 100 mg/mL of streptomycin, 2 mmol/L of L-glutamine, and 30 mmol/L of HEPES (all from Life Technologies GIBCO, BRL, Gaithersburg, MS). Cells were cultured either stimulated with 10 *μ*g/mL of SEA (*Schistosoma* egg antigen) or without stimulation to assess cytokine production at 37°C in an atmosphere containing 5% CO_2_ for 16 h. After incubation, the cells were stained with fluorochrome-conjugated antibody and acquired using flow cytometry (FACSCanto, BD Biosciences, San Jose, CA) as described below.

### 2.5. Flow Cytometry

**Figure 1:**
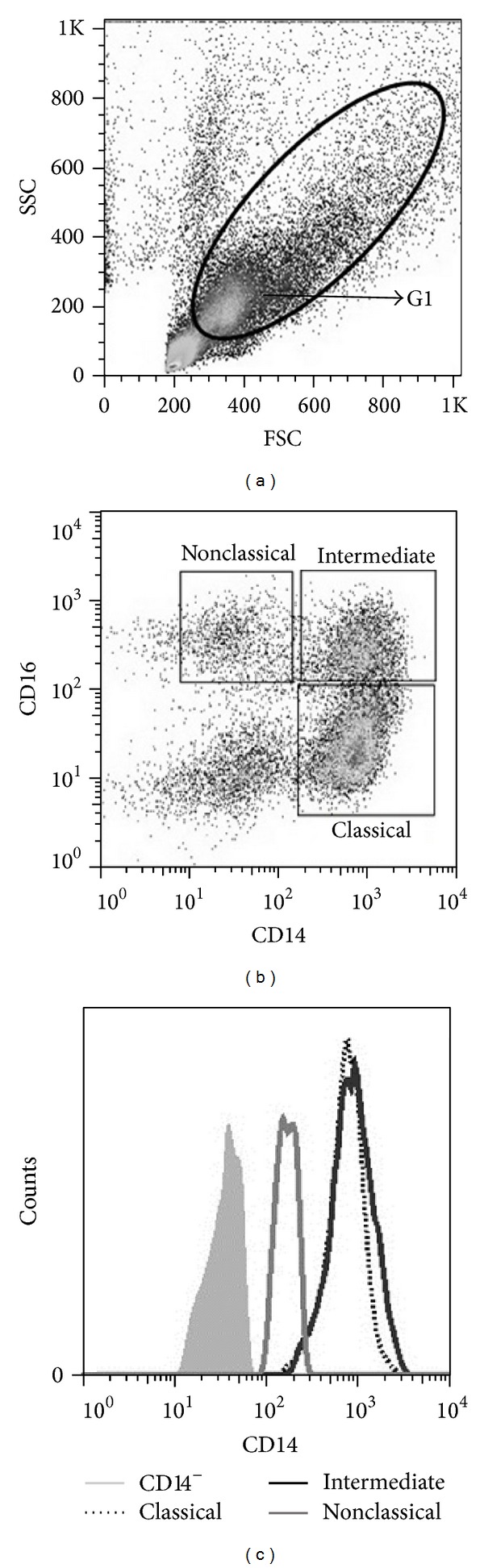
The monocyte population was defined by nonspecific fluorescence from the forward scatter (FSC) and side scatter (SSC) as parameters of cell size and granularity, identifying the monocyte population, region 1 (G1) (a). Strategy for classification of monocyte subsets through the expression of CD14 and CD16 (b). A representative histogram of CD14 expression in monocyte subsets (c).

PBMCs (3 × 10^5^) obtained by a Ficoll-Hypaque gradient were incubated 10 *μ*g/mL of SEA for 16 h, 37°C, and 5% of CO_2_. During the last 4 h of culture, Brefeldin A (10 *μ*g/mL; Sigma, St. Louis, MO), which impairs protein secretion by the Golgi complex, was added to the cultures. Afterwards, the cells were stained with PERCPCy5.5-labeled antibody conjugated with CD14 (anti-CD14 PERCPCy5.5; clone 61D3), anti-CD16 FITC (clone CB16), anti-IL-10R PE (polyclonal), anti-IL-4R*α* PE (clone hIL-4R-M57), and anti-HLA-DR PE (clone LN3) and then washed in PBS and fixed in 4% formaldehyde for 20 min at room temperature. Intracellular staining was performed with anti-IL-12 PE (clone C8.6) mAbs, anti-IL-10 APC (clone JES3-19F1), anti-TGF-*β* APC (clone 9016), anti-IL-6 APC (clone MQ2-13A5), and anti-TNF-*α* PECy7 (MAb11), all antibodies from eBioscience. The monocyte population was defined by nonspecific fluorescence from the forward scatter (FSC) and side scatter (SSC) as parameters such as cell size and granularity, respectively. Monocyte area corresponded to the specific region graph: region 1 (G1) (Figure 1(a)). A total of 100,000 events were acquired for all experiments.

### 2.6. Analysis of FACS Data

The frequency of positive cells was analyzed using the program Flow Jo (Tree Star, USA). The monocytes subsets were selected based on the expression of CD14 and CD16 (Figure 1(b)). A representative histogram of CD14 expression in monocytes subsets is shown in Figure 1(c). We also evaluated the expression of CD56 in the population of monocytes and it was negative to this NK cell marker (data not shown). Limits for the quadrant markers were set based on negative populations and controls isotype (data not shown). Data were expressed as mean fluorescence intensity (MFI) parameter.

### 2.7. Statistical Analysis

Statistical analysis and the graphical representation were performed using the computer program Graphpad PRISM 5.0 software (La Jolla, CA, USA). Between-group comparisons were done using parametric and nonparametric methods as appropriate (ANOVA and Kruskal Wallis test). All statistical tests were two-tailed and the statistical significance was established at the 95 percent confidence interval and significance was defined to *P* < 0.05. The Ethical Committee of Climério de Oliveira Maternity of the Federal University of Bahia, Brazil, approved the present study, and informed consent was obtained from all participants or their legal guardians.

## 3. Results

### 3.1. Monocyte Populations in Subjects with Different Degrees of Periportal Fibrosis due to Schistosomiasis

We evaluated the frequency of different subpopulations of monocytes and the activation status of these cells. The frequency of intermediate (CD14^++^CD16^+^) and nonclassical (CD14^+^CD16^++^) monocytes in cultures stimulated with SEA was higher (17% (7.72%–32.3%), 11.1% (2.71%–27.4%), resp.) in the group of individuals with incipient fibrosis when compared with the group without fibrosis (13.8% (3.88%–22.8%), and 7.56% (1.88%–24%); resp.). There was no significant difference in the frequency of classical monocytes (CD14^++^CD16^−^) among subjects with different degrees of periportal fibrosis (without fibrosis: without stimulus (WS) 56.3% (32.3%–73.9%), SEA 50.1% (32%–73.9%); incipient fibrosis: WS 45.4% (24.2%–71.6%), SEA 46.1% (22.3%–74.6%); moderate to severe fibrosis: WS 40.1% (19.2%–73.8%), SEA 46.5% (15.3%–75.6%). Furthermore, we observed that independently of the degree of fibrosis, the frequency of classical monocytes was higher in nonstimulated cultures (56.3% (32.3%–73.9%)) and also in cultures stimulated with SEA (50.1% (32%–73.9%); *P* < 0.0001) as compared to the frequency of intermediate (WS: 14.35% (6.3%–23.4%) and SEA: 13.8% (3.88%–22.8%); *P* < 0.0001) and nonclassical monocytes (WS: 5.36% (2.24%–26.2%) and SEA: 7.5% (1.88%–24%), *P* < 0.0001).

**Figure 2:**
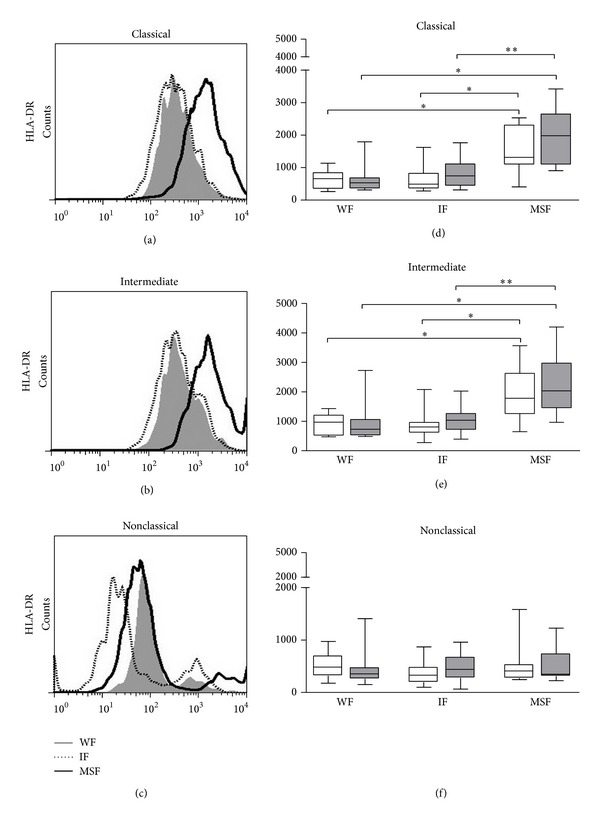
Representative histogram of HLA-DR expression (mean fluorescence intensity, MFI) on monocytes of patients with periportal fibrosis secondary to schistosomiasis ((a)–(c)). Expression of HLA-DR on classical (CD14^++^CD16^−^), intermediate (CD14^++^CD16^+^), and nonclassical (CD14^+^CD16^++^) monocytes, respectively, in cultures without stimulation (white bar) and stimulated with 10 *μ*g/mL of SEA (gray bar) ((d)–(f)). Without fibrosis (WF), incipient fibrosis (IF), and moderate to severe fibrosis (MSF). *P < 0.05 and **P < 0.005 (Kruskal-Wallis).

The expressions of HLA-DR on monocytes from individuals with different degrees of periportal fibrosis were evaluated. We observed that in the group of individuals with moderate to severe fibrosis the expression of HLA-DR on monocytes was higher both in classical (Figure 2(d)) (WS MFI = 1318 (404–2531), SEA MFI = 1981 (906–3419)), and intermediate monocytes (Figure 2(e)) (WS MFI = 1782 (648–3563), SEA MFI = 2033 (965–4198)) compared to the group of individuals with incipient fibrosis (classical: WS MFI = 492 (278–1623), SEA MFI = 747.5 (313–1766) and intermediate: WS MFI = 807 (278–2079), SEA MFI = 1034 (392–2022)) and without fibrosis (classical: WS MFI = 659 (263–1138), SEA MFI = 533 (315–1791), and Intermediate: WS MFI = 970 (474–1430), SEA MFI = 733 (486–2733)). However, there was no significant difference in the expression of HLA-DR on nonclassical monocytes among subjects with different degrees of periportal fibrosis (Figure 2(f)). The representative histogram for HLA-DR is shown in Figures 2(a)–2(c).

### 3.2. Expression of Profibrotic and Proinflammatory Molecules in Monocyte Subsets in Subjects with Different Degrees of Periportal Fibrosis Secondary to Schistosomiasis

The subsets of monocytes from individuals with different degrees of periportal fibrosis were evaluated regarding the expression of the profibrotic markers such as IL-4R*α* and TGF-*β* and proinflammatory cytokines such as IL-6 and TNF-*α*.

**Figure 3:**
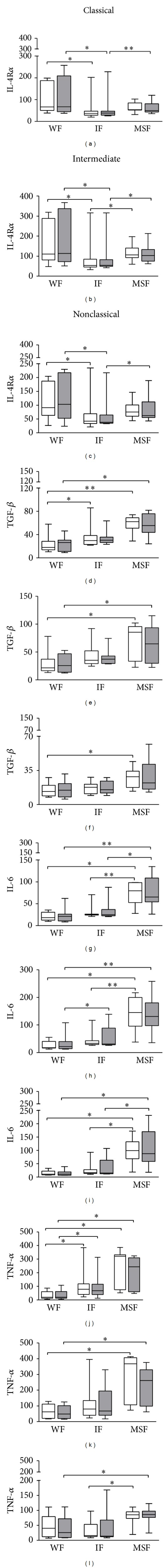
Mean fluorescence intensity (MFI) expression of profibrotic IL-4R*α* ((a)–(c)) and TGF-*β* ((d)–(f)) and proinflammatory IL-6 ((g)–(i)) and TNF-*α* ((j)–(l)) molecules in classical (CD14^++^CD16^−^), intermediate (CD14^++^CD16^+^), and nonclassical (CD14^+^CD16^++^) monocytes, of patients with different degrees of periportal fibrosis secondary to schistosomiasis. Cultures without stimulus (white bar) and cultures stimulated with 10 *μ*g/mL SEA (gray bar). Without fibrosis (WF), incipient fibrosis (IF), and moderate to severe fibrosis (MSF). *P < 0.05 and **P < 0.005 (Kruskal-Wallis).

The *α* receptor expression of IL-4 in the group of patients without fibrosis was higher in classical (Figure 3(a)) (WS MFI = 66.5 (38.5–199), SEA MFI = 67.15 (37.4–257)), intermediate (Figure 3(b)) (WS MFI = 110 (48.2–319), SEA MFI = 113.5 (50.9–367)), and nonclassical monocytes (Figure 3(c)) (WS MFI = 90.7 (26.3–205), SEA MFI = 103.4 (24.1–230)) compared with the group of subjects with incipient fibrosis (classical: WS MFI = 36.7 (19.5–202), SEA MFI = 38.35 (26–228); intermediate: WS MFI = 53.6 (32.5–316), SEA MFI = 54.1 (42–316); and nonclassical: WS MFI = 42.25 (21.2–235), SEA MFI = 38 (32.8–217)). The expression of this receptor was also higher in the three subpopulations of monocytes from patients with moderate to severe fibrosis (classical: SEA MFI = 49.9 (35.5–120); intermediate: WS MFI = 106 (59.7–197), SEA MFI = 103 (62.1–213), and nonclassical: SEA MFI = 62.6 (42.9–189)) compared with the group of individuals with incipient fibrosis. In the classical (Figure 3(a)) and nonclassical monocytes (Figure 3(c)) this difference was only observed in cultures stimulated with SEA.

The expression of TGF-*β* in the group of subjects with moderate to severe fibrosis was higher in classical (WS MFI = 62.25 (29.1–74,1) SEA MFI = 55.5 (24.1–81.8)) (Figure 3(d)), intermediate (WS MFI = 85.4 (22.5–102), SEA MFI = 64.7 (22.4–115)) (Figure 3(e)), and nonclassical monocytes (WS MFI = 28.5 (13.7–44.2)) (Figure 3(f)) compared with the group of patients without fibrosis (classical: WS MFI = 18.05 (10.4–58.2), SEA MFI = 26.2 (8.9–46.2); intermediate: WS MFI = 21.6 (13–77.9), SEA MFI = 25.6 (12.2–52.8); and nonclassical: WS MFI = 13.45 (7.6–27.7)). Furthermore, the expression of TGF-*β* in classical monocytes in cultures without antigenic stimulation was higher in the group of individuals with incipient fibrosis (MFI = 29.8 (21.9–86)) when compared to individuals without fibrosis (MFI = 18.05 (10.4–58.2)) (Figure 3(d)).

The expression of IL-6 (Figures 3(g)–3(i)) was higher in all three subpopulations of monocytes from the group of patients with moderate to severe fibrosis (classical: WS MFI = 79.8 (28–98.6), SEA MFI = 65.45 (26.5–135), intermediate: WS MFI = 145 (38.4–217), SEA MFI = 131 (36–258), and nonclassical: WS MFI= 99.15 (18.5–172), SEA MFI = 87.6 (17.9–231)) compared with the group of patients without fibrosis (classical: WS MFI = 18.35 (9.75–36), SEA MFI = 21.4 (8.6–62.5); intermediate: WS MFI = 17.9 (12.6–55.5), SEA MFI = 21.85 (12.3-108); and nonclassical: WS MFI = 10.8 (7.23–32.8), SEA MFI = 10.6 (6.63–39.2)) and with incipient fibrosis (classical: WS MFI = 25.4 (21.1–70.9), SEA MFI = 25.2 (20.7–87.6); intermediate: WS MFI = 33.1 (26.1–117); and nonclassical: WS MFI = 16.2 (10.1–93), SEA MFI = 11.1 (15.2–107)). We also observed that the expression of IL-6 in intermediate monocytes was higher in the group of individuals with incipient fibrosis (SEA MFI = 31.2 (26–139], related to individuals without fibrosis (SEA MFI = 21 85 (12.3–108)) (Figure 3(h)).

Regarding the expression of TNF-*α*, we observed that the group of subjects with moderate to severe fibrosis showed higher expression of this cytokine in classical (Figure 3(j)) (WS MFI = 321 (52–386), SEA MFI = 244 (47.4–324)), intermediate (Figure 3(k)) (WS MFI = 368 (73.1–413), SEA MFI = 261 (62.8–377)), and nonclassical monocytes (Figure 3(l)) (SEA MFI = 85.6 (24.3–123)), when compared to individuals without fibrosis (classical: WS MFI = 20.5 (5.32–86.9), SEA MFI = 20.5 (4.1–108); intermediate: WS MFI = 62.7 (17.4–129), SEA MFI = 48.7 (14.7–125); and nonclassical SEA MFI= 25.65 (8–112)). There was also a higher expression of TNF-*α* in nonclassical monocytes, in cultures without antigen stimulation, from the group of patients with moderate to severe fibrosis (MFI = 85.2 (19.6–111)) (Figure 3(l)) compared to individuals with incipient fibrosis (MFI = 15.7 (8.5–97.6)). Additionally, there was an increased expression of TNF-*α* in the classical monocytes of patients with incipient fibrosis (WS MFI = 78.8 (22.4–384), SEA MFI = 67.9 (12.9–313)) when compared with individuals without fibrosis (Figure 3(j)).

### 3.3. Regulatory and Antifibrotic Molecules in Monocyte Subsets in Subjects with Different Degrees of Periportal Fibrosis Secondary to Schistosomiasis

**Figure 4:**
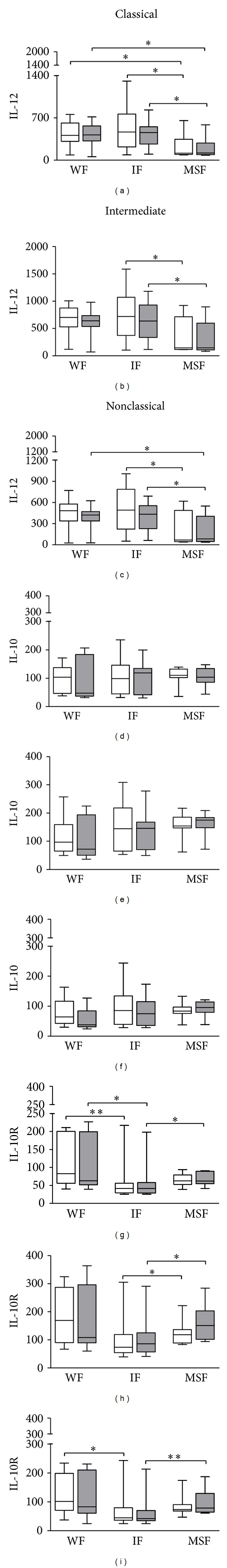
Mean fluorescence intensity (MFI) expression of antifibrotic IL-12 ((a)–(c)) and regulatory IL-10 ((d)–(f)) and IL-10R ((g)–(i)) molecules in classical (CD14^++^CD16^−^), intermediate (CD14^++^CD16^+^), and nonclassical (CD14^+^CD16^++^) monocytes of patients with different degrees of periportal fibrosis secondary to schistosomiasis. Cultures without stimulation (white bar) and cultures stimulated with 10 *μ*g/mL of SEA (gray bar). Without fibrosis (WF), incipient fibrosis (IF), and moderate to severe fibrosis (MSF). *P < 0.05 and **P < 0.005 (Kruskal-Wallis).

The expression of IL-12, IL-10, and IL-10R was evaluated in monocytes of schistosomiasis patients. The expression of IL-12 was higher in the classical (Figure 4(a)) (WS MFI = 464 (87.5–1310) and SEA MFI = 456.5 (97.6–831)), intermediate (Figure 4(b)) (WS MFI = 722.5 (104–1589) and SEA MFI = 635 (117–1180)), and nonclassical monocytes (Figure 4(c)) (WS MFI = 492.5 (52.1–1006) and SEA MFI = 434 (62.1–690)) of individuals with incipient fibrosis compared with the group of subjects with moderate to severe fibrosis (classical: WS MFI = 115.5 (85.4–656) and SEA MFI = 117.5 (81–584); intermediate: WS MFI = 144 (113–922) and SEA MFI = 145.5 (83.8–896), and nonclassical: WS MFI = 67.45 (39.7–618) and SEA MFI = 84.8 (37.5–550)). Furthermore, we observed that in the group of individuals without fibrosis the expression of IL-12 was higher in both classical (WS MFI = 408 (86.2–757) and SEA MFI = 416 (57.2–716)) and the nonclassical monocytes (SEA MFI = 422 (28.4–624)) when compared with the group of patients with moderate to severe fibrosis. In nonclassical monocytes this difference was only observed in the presence of SEA.

In relation to the expression of regulatory molecule IL-10 in these monocytes there was no significant differences among subjects with different degrees of periportal fibrosis (Figures 4(d)–4(f)).

The expression of IL-10R, however, was higher in classical (Figure 4(g)) (SEA MFI = 62.3 (41.6–90.8)), intermediate (Figure 4(h)) (WS MFI = 118 (83.2–291) and SEA MFI = 151 (93.8–284)), and nonclassical monocytes (Figure 4(i)) (SEA MFI = 78.6 (61.3–187)) in the group of patients with moderate to severe fibrosis when compared with the group of subjects with incipient fibrosis (classical: SEA MFI = 41.75 (25.2–198); intermediate: WS MFI = 73.8 (38.8–305) and SEA MFI = 85.75 (40.8–291); and nonclassical: SEA MFI = 42.35 (24.6–213)). Furthermore, in the group of individuals without fibrosis the expression of IL-10R was higher in classical (WS MFI = 82.6 (40–211) and SEA MFI = 63.1 (39.7–227)) and nonclassical monocytes (WS MFI = 101.4 (37.8–234)) compared to the group of individuals with incipient fibrosis (classical: WS MFI = 41.45 (25.4–217) and SEA MFI = 41.75 (25.2–198) and nonclassical: WS MFI = 44.8 (25–243)).

## 4. Discussion

This study aimed to characterize the phenotype of monocytes of patients with different degrees of periportal fibrosis secondary to schistosomiasis. The mean age of individuals with moderate to severe fibrosis was higher when compared with individuals without fibrosis. This is in agreement with the literature [18, 21] and may be explained by constant reexposure to the parasite, or by the slow process of fibrosis development [22]. Parasite burden and gender distribution were similar among groups of patients with different degrees of fibrosis. However, other studies from our group have shown that individuals without fibrosis have a higher parasite burden as compared to the other groups [18, 20]. A possible explanation for this observation is that chronic infection can lead to intestinal fibrosis, which may impair the migration of eggs to the intestinal lumen and thereby decrease egg count in parasitological exams [23]. Other studies have examined the role of gender in the development of periportal fibrosis, noting an overall male bias in susceptibility [24, 25].

We did not observe a significant difference between groups of individuals with respect to liver size, likely because in those with moderate to severe periportal fibrosis, the liver parenchyma was not severely compromised. It is well known that the liver size decreases with the progression of fibrosis [19, 26–28].

We observed, however, that in patients with moderate to severe fibrosis spleen size was larger as compared to the other groups. Portal vein diameter was also higher in subjects with moderate to severe fibrosis as compared to patients without fibrosis. These findings are in agreement with other studies [29, 30].

In recent years, studies have emphasized the important role of monocytes in the inflammatory process associated with hepatic fibrosis in experimental models [31–34].

Wong et al. evaluated characteristics of classical monocytes, intermediate, and nonclassical through the gene expression profiling and observed that classical monocytes expressed genes involved in tissue repair function, the intermediate monocytes expressed genes for MHC class II [35], and nonclassical monocytes with genes involved in cytoskeleton rearrangement which may be responsible for its high motility observed* in vivo* [36]. Little is known about the role of monocytes in the pathology of human schistosomiasis, and this study aimed to characterize the monocyte subsets (classical, intermediate, and nonclassical) in schistosomiasis patients with different degrees of periportal fibrosis.

HLA-DR expression on monocytes is important to antigen presentation to T cells. We observed that in patients with moderate to severe fibrosis the expression of HLA-DR on classical and intermediate monocytes was higher as compared to the patients with incipient fibrosis and without fibrosis. However, there was no significant difference in the expression of HLA-DR on nonclassical monocytes among the groups. These results indicate that classical and intermediate monocytes might participate in periportal fibrosis development in schistosomiasis through antigen presentation and subsequently, in T cell activation. Studies using the new classification of monocytes in humans are rare. Hudig et al. observed that the different phenotypes of monocytes from human individuals is heterogeneous and may alter depending on the disease model [37]. However, there are some studies in the literature showing a low expression of HLA-DR by nonclassical monocytes, suggesting that this population has a patrolling profile and lower capacity to present antigens to T cells [35, 36, 38]. Furthermore, study of our group also observed that the addition of* Schistosoma mansoni* antigen rSm29 in PBMC cultures in the presence of soluble* Leishmania braziliensis* antigen (SLA) decreases the expression of HLA-DR in nonclassical monocytes from patients with cutaneous leishmaniasis [16].

In order to better understand the mechanisms involved in the development of periportal fibrosis in schistosomiasis, molecules of profibrotic and proinflammatory profiles were also evaluated in different subpopulations of monocytes.

The expression of IL-4R*α* in the three subpopulations of monocytes was higher in patients without fibrosis and moderate to severe fibrosis when compared with individuals with incipient fibrosis. In experimental models, IL-4 and IL-13 cytokines are responsible for inducing an alternative activation of monocytes through binding to the *α* receptor of IL-4. The signaling through the IL-4R*α* induces the expression of arginase, an enzyme responsible for the conversion of L-arginine in proline. The proline is an essential amino acid which is involved in collagen production and development of fibrosis [39]. In experimental models of* S. mansoni* infection it has been shown that the responsiveness through the IL-4R*α* is important to granuloma formation and survival of the host during infection [40, 41]. However, there are few studies reporting the role of IL-4R*α* in human monocytes and the role of this molecule in schistosomiasis remains controversial.

The expression of TGF-*β* in the three subpopulations of monocytes was also higher in subjects with moderate to severe fibrosis compared to individuals without fibrosis. This cytokine induces fibroblast proliferation and collagen deposition, suggesting its potential role in the establishment of fibrosis [42–44]. In the study conducted by Souza et al. there was no significant difference in serum levels of TGF-*β* in schistosomiasis patients with different degrees of periportal fibrosis [18]. In addition, other studies have not found differences in the levels of TGF-*β* in PBMCs cultures stimulated with SEA among groups of individuals with different degrees of periportal fibrosis [20, 21]. Possibly the expression of TGF-*β* by human monocytes is associated with fibrosis, while the production of this cytokine by lymphocytes is not essential. Study performed by Kanzler et al. (2001) supports the role of TGF-*β* in liver fibrogenesis in patients with hepatitis C infection. The authors propose that TGF-*β* predicts clinical disease progression [45]. However, Li et al. observed that local production of TGF-*β* by regulatory T cell appeared to have a protective role in fibrogenesis in individual with hepatitis C virus infection [46].

Following the same pattern of cytokine production, the expression of the inflammatory cytokine IL-6 was higher in all three subpopulations of monocytes from individuals with moderate to severe fibrosis as compared to individuals with incipient fibrosis and without fibrosis and was also higher in intermediate monocytes of individuals with incipient fibrosis as compared to individuals without fibrosis. Khalil et al. (1996) reported an increased production of IL-6 during the course of* S. mansoni* infection and granuloma formation in an experimental model, which may indicate the involvement of this cytokine in liver pathology [47]. Another experimental study reported a high production of IL-6 by cultures of macrophages isolated from granulomas in response to SEA antigen [48]. IL-6 is a phase acute cytokine associated to fibrogenesis and collagen deposition [49–52], and Fuster et al. observed that IL-6 was strongly associated with liver fibrosis in HIV-infected patients with alcohol problems, and this cytokine may be a useful predictive marker for liver fibrosis for these patients [53].

Recently, it has been shown that the combination of TGF-*β* and IL-6 is crucial for the differentiation of naive T cells into Th17 cells [54–57]. IL-17 is a cytokine involved in the development of several diseases, recruiting neutrophils and macrophages to inflammatory sites [58]. The participation of IL-17 in the pathogenesis of schistosomiasis has been described in experimental models [59–61]. In our study, we observed high expression of IL-6 and TGF-*β* in monocytes of individuals with moderate to severe fibrosis. These cytokines may contribute to the induction of IL-17 and consequently lead to a worsening of the disease. Souza et al., however, found no difference in the levels of IL-17 in supernatants of PBMCs cultures stimulated with SEA between groups of individuals with different degrees of periportal fibrosis [18].

The expression of TNF-*α* was higher in monocytes of patients with moderate to severe fibrosis as compared to individuals without fibrosis or with incipient fibrosis. High levels of TNF-*α* produced by PBMC stimulated with* Schistosoma* antigens or in nonstimulated cells have been found in patients with periportal fibrosis [10, 18, 22, 62]. However, other studies have not observed any differences in TNF-*α* levels in supernatants of PBMC stimulated with SEA in patients with different degrees of fibrosis [20, 21]. In experimental studies TNF-*α* seems to be essential to liver fibrosis development [63]. In chronic hepatitis B virus infection CD16^+^ subset of monocytes produces high levels of TNF, suggesting that this subset of monocytes may be closely related to liver damage in these HBV-infected patients [64]. Our study showed that monocytes express TNF-*α*, independent of their subpopulation, and this may be important for the development of fibrosis.

Besides the inflammatory and profibrotic role of monocytes or macrophages, these cells are also essential in preventing fibrosis in experimental models [65, 66].

In our study, the expression of IL-12 by monocytes was higher in individuals with incipient fibrosis and without fibrosis when compared to individuals with moderate to severe fibrosis. Some studies have shown that the granulomatous inflammation and hepatic fibrosis in experimental models of* S. mansoni* can be prevented by the addition of IL-12, a key cytokine that induces the Th1 immune response [67–69]. Hoffmann et al. showed that the deviation of the immune response to the Th1 type is required to reduce granuloma size and prevent hepatic fibrosis [63]. As monocytes of patients with moderate to severe fibrosis in our study showed a low expression of IL-12, these patients might present an impaired Th1 response and insufficient control of fibrosis development.

IL-10 is a cytokine with modulatory functions; however, we found no significant difference in the expression of IL-10 in monocytes from individuals with different degrees of periportal fibrosis. Some authors have shown elevated levels of this cytokine in PBMC from patients with severe periportal fibrosis [20], while others have found low levels of this cytokine in these patients [21], or no significant difference in the levels of IL-10 among groups of patients [18]. Experimental studies of double IL-10 and IL-12 knockouts resulted in severe fibrosis development [69, 70].

The expression of IL-10 receptor in this study, however, was higher in monocytes of patients with moderate to severe fibrosis and without fibrosis compared to individuals with incipient fibrosis. This was unexpected, since Herbert et al. found that the administration of anti-IL-10R monoclonal antibody in mice infected with* S. mansoni* significantly increased the production of IL-4, IFN-*γ*, TNF-*α*, and IL-17, as well as the size of hepatocellular damage [71].

The expression of IL-4R*α* and IL-10R by monocytes of patients without fibrosis might contribute to the anti-inflammatory profile of monocytes in these individuals. Moreover, in a recent study from our group we observe that the lymphocytes of individuals with moderate and severe fibrosis had a lower expression of the molecule activation CD28 and low expression of regulation markers, such as CTLA-4 and CD25^high^, suggesting the absence of regulation in lymphocytes of these individuals with degrees higher of fibrosis [72].

## 5. Conclusion

Taken together, our results indicate that monocyte subpopulations of patients with moderate to severe periportal fibrosis participate in the immunopathogenesis of the disease, since they express high levels of proinflammatory and profibrotic cytokines in combination with a low expression of regulatory molecules.

## Figures and Tables

**Table 1:** Characteristics of the studied population.

Fibrosis	Without fibrosis (*n* = 17)	Incipient fibrosis (*n* = 15)	Moderate to severe (*n* = 8)	*P*
Age (years)* (mean ± SD)	30 ± 13	37 ± 12	46 ± 14	*P* < 0.05^a^
Male gender *n* (%)**	6 (35.3)	5 (33.3)	2 (25)	*P* > 0.05
Parasite burden (epg)*** Median (min–max)	72 (24–392)	72 (24–600)	66 (24–192)	*P* > 0.05
^ 1^Liver size (cm)*	9.5 ± 1.3	10.3 ± 1.5	10.9 ± 2.2	*P* > 0.05
^ 2^Spleen size (cm)*	8.4 ± 1.7	8.3 ± 1.4	10.9 ± 2.4	*P* < 0.05^a.b^
Portal vein diameter (mm)*	6.8 ± 3.0	7.9 ± 3.3	11.3 ± 5.0	*P* < 0.05^a^

*ANOVA; **chi-square; ***Kruskal-Wallis; ^a^moderate to severe fibrosis versus without fibrosis.

^b^Moderate to severe fibrosis versus incipient fibrosis.

^1^Measured by midclavicular line.

^2^The largest diameter of the organ measured by USG.
